# Calcium Prevents Enhanced Degradation of Factor VIII in the Condition of Motion

**DOI:** 10.3390/biology12111388

**Published:** 2023-10-30

**Authors:** Haim Cohen, Anat Keren-Politansky, Yonatan Crispel, Chen Yanovich, Keren Asayag, Yona Nadir

**Affiliations:** 1 Thrombosis and Hemostasis Unit, Department of Hematology, Rambam Health Care Campus, Haifa 31096, Israel; haimge1@walla.co.il (H.C.); anat_keren@rambam.health.gov.il (A.K.-P.); y_crispel@rambam.health.gov.il (Y.C.); hionobich@gmail.com (C.Y.); kerenasayag@walla.co.il (K.A.); 2 The Rappaport Faculty of Medicine, Technion, Haifa 31096, Israel

**Keywords:** factor VIII, calcium ions, movement, hemophilia, degradation of factor VIII, calcium supplement

## Abstract

**Simple Summary:**

In the present work, calcium was found to prevent degradation of factor VIII during intermittent motion. In addition, calcium supplementation in drinking water consumed by mice increased factor VIII in blood and striated muscle. The clinical relevance of this work is that oral calcium may be evaluated as a potential supportive therapy in hemophilia patients. Another aspect is that food additives with calcium may increase factor VIII level, which is known to contribute to both arterial and venous thrombosis.

**Abstract:**

**Background:** Hemophilia A and B induce recurrent bleeding episodes, mainly in skeletal muscles and joints that are in intermittent motion. We have previously demonstrated that intermittent motion contributes to increased degradation of factors VIII and IX. **Objectives:** Given that calcium ions are known to enhance factor VIII–von Willebrand factor (vWF) interaction, the present study has investigated the role of these ions on factors VIII and IX in the condition of motion. **Methods:** The effects of calcium ions were assessed using purified proteins via Western blot, factor VIII activity, immunocytochemistry, and in Institute of Cancer Research (ICR) mice with no specific genetic background. **Results:** Calcium was found to prevent degradation of plasma-derived factor VIII but not that of factor IX, during intermittent motion. Calcium levels in the microcirculation of mouse striated muscles were elevated following movement, enabling prevention of factor VIII degradation in normal physiology. Calcium supplementation in drinking water increased factor VIII levels in blood and striated muscles of ICR mice during movement. **Conclusions:** calcium ions decrease factor VIII degradation in the condition of motion. Further research on the impact of calcium salt oral supplementation on hemophilia patients is warranted.

## 1. Introduction

Hemophilia A and B are due to deficiency of coagulation factors VIII and IX, respectively [1,2]. The estimated prevalence of these two rare X-linked inherited bleeding disorders in the USA is 12 per 100,000 males for hemophilia A, and 3.7 per 100,000 males for Hemophilia B [3]. These patients suffer from recurrent bleeding, primarily in skeletal muscles and joints, which are organs in intermittent motion [4,5]. Patients with severe hemophilia are typically diagnosed in the first two years of life subsequent to soft tissue bleeding. Without prophylactic treatment, these individuals may experience 2–5 monthly spontaneous bleeding events, mostly joint bleeding and deep-muscle hematomas [6]. Replacement therapies with coagulation factor concentrates are used to treat and prevent bleeding [2]. These clotting factor replacement therapies differ in half-life length. The main complication of factor replacement is the development of neutralizing antibodies to the clotting factor [2,7]. Non-replacement treatments that have been developed as additional options for prophylaxis include rebalancing hemostatic agents that antagonize the natural anticoagulant proteins with a reduction in tissue-factor pathway inhibitor, protein C, or anti-thrombin. In addition to these treatments, emicizumab, a bispecific antibody that imitates the function of factor VIII, was approved for prophylaxis treatment for hemophilia A patients of all ages, with and without factor VIII inhibitors. Gene therapy studies are continuing to prove efficacy and safety [7]. Due to breakthrough bleeding, surgery, and trauma, hemophilia patients using prophylaxis treatment need additional factor replacement. Currently, research in the field of hemophilia replacement therapy is ongoing to improve unmet needs. 

We have recently suggested a potential mechanism underlying the increased tendency of muscle and joint bleeding, based on the finding that intermittent motion induces increased degradation of factor VIII and IX [8]. We have also demonstrated a protective effect of factor VIII on factor IX level, and vice versa [8]. Calcium ions had earlier been reported to stabilize both native and thrombin-activated factor VIII–von Willebrand factor (vWF) complexes using in vitro assays [9,10]. To that end, the present study aimed to explore if the stabilization effect of calcium on factor VIII persists in the condition of motion, and if oral supplementation of calcium to mice could prevent enhanced factor VIII degradation during movement.

## 2. Materials and Methods

### 2.1. Reagents and Antibodies

Human thrombin was purchased from Siemens (Munich, Germany). Plasma-derived Factor VIII Hemofil-M was obtained from Baxter (CA, USA) and plasma-derived Factor IX Rerplenine–VF was purchased from Bio Products Laboratory (Hertfordshire, UK). All coagulation factors were dissolved in double-distilled water. A polyclonal anti-factor VIII light chain (H-100) was purchased from Santa Cruz Biotechnology, Inc. (Heidelberg, Germany). Polyclonal anti-factor IX was acquired from Novus Biologicals Europe (Abingdon, UK) and Alizarin Red powder was purchased from Sigma (St. Louis, MO, USA). 

### 2.2. Mouse Model

The study was approved by the Technion Ethics Committee for Animal Research (approval code IL0510319), and the procedures followed were in accordance with institutional guidelines. Institute of Cancer Research (ICR) mice with no specific genetic background were used in the study. All the experiments were performed in seven- to eight-week-old male mice in order to avoid hormonal effects on factor VIII. 

### 2.3. Coagulation Assays

Levels of coagulation factor VIII activity were evaluated using a 1-stage assay with factor VIII deficient plasma (Diagnostica Stago, Asnières sur Seine Cedex, France) and determined on a Sysmex CA1500 analyzer (Siemens Healthcare Diagnostics, Marburg, Germany). Detailed description of the 1-stage assay that was used to determine factor VIII levels in the plasma is presented in an article by Malar et al. [11]. 

### 2.4. SDS-Polyacrylamide Gel Electrophoresis (PAGE) and Immunoblot Analysis

Proteins were subjected to 10% SDS-PAGE and transferred to polyvinylidene fluoride membrane (Bio-Rad, Maylands, CA, USA). The membrane was probed with the appropriate antibody followed by horseradish peroxidase-conjugated secondary antibody (Jackson ImmunoResearch, West Grove, PA, USA) and chemiluminescence substrate (Pierce, Rockford, IL, USA). Rabbit anti-factor VIII light chain, or rabbit anti-factor IX diluted to 1/2000 was used, as previously described [12,13].

### 2.5. Immunohistochemistry

Staining of formalin-fixed, paraffin-embedded 5-micron sections of muscle was performed. Slides were deparaffinized with xylene, rehydrated, and endogenous peroxidase activity was quenched for 30 min using 3% hydrogen peroxide in methanol. The slides were then subjected to antigen retrieval by boiling (20 min) in a 10 mM citrate buffer, pH 6. Slides were further incubated with 10% normal goat serum in PBS for 60 min to block non-specific binding followed by incubation (20 h, 4 °C) with anti-factor VIII (1:250 dilution). Slides were then extensively washed with PBS containing 0.01% Triton X-100 and incubated with a secondary antibody, according to the manufacturer’s instructions (Envision kit; Dako, Glostrup, Denmark). Following additional washes, color was developed with the AEC reagent (Sigma, St. Louis, MO, USA) and sections were counterstained with hematoxylin and mounted. Analyses of tissues’ immunohistochemistry results were performed by two of the authors, who were unaware of the slide allocation. Discrepancies in the analyses were reconciled following assessment by a third reviewer. Five high-power fields were evaluated in each stained slide. Staining intensity was scored as follows: 0, no staining; 1, weak intensity; 2, moderate intensity; and 3, marked intensity.

### 2.6. Alizarin Red Staining

According to the manufacturer’s recommendations, staining of formalin-fixed, paraffin-embedded 5-micron sections of muscle was performed. Slides were deparaffinized with xylene, rehydrated, and endogenous peroxidase activity was quenched for 30 min using 3% hydrogen peroxide in methanol. Slides were then washed in distilled water and stained with the Alizarin Red Solution, added for 2 min. Then, the slides were dehydrated in acetone (20 dips), dipped in acetone–xylene (1:1), solution (20 dips), cleared in xylene, and mounted in a synthetic mounting medium.

### 2.7. Statistical Analysis

Data were evaluated using SPSS software for Windows, version 13.0 (SPSS Inc., Chicago, IL, USA). Statistics were calculated using the non-parametric Mann–Whitney U-test. Values were reported as median and range or mean ± SEM, as indicated. The significance level was set at *p* < 0.05.

## 3. Results

### 3.1. Calcium Prevents Degradation of Factor VIII during Intermittent Motion

**Figure 1:**
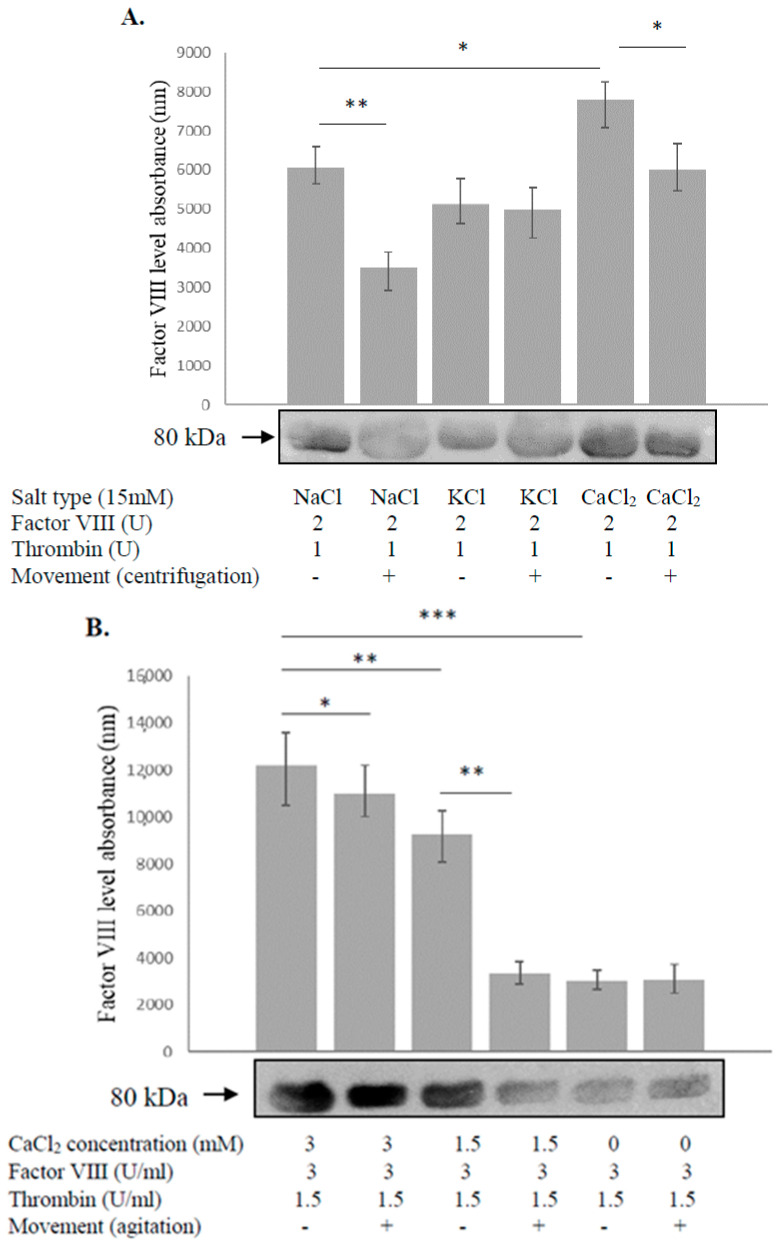
Calcium prevents degradation of factor VIII during motion. (**A**). Factor VIII, which included thrombin dissolved in the indicated salt solutions, was set in motion via 10 min centrifugation (500 g) at room temperature, and then the level of factor VIII was measured via Western blot. Comparison of the effects elicited by each of the above salts, dissolved at the same concentration in double-distilled water (DDW), demonstrated that only calcium prevented the degradation of factor VIII. The protective effect was preserved even when the solutions were centrifuged. As all the assessed salts have Cl^−^ ions, the comparison indicates that Ca^+2^ ions and not Cl^−^ ions are responsible for the stabilization effect. Calcium dissolved in DDW at a physiologic plasma concentration (1.5–3 mM) still prevented degradation of factor VIII in a dose-dependent manner, and the effect was maintained following agitation for 10 min at 30 RPM (**B**). Relative protein levels in Western blots were quantified via densitometry analysis (**upper panel**). Assays were conducted in triplicate. The results represent mean and range. * *p* < 0.05, ** *p* < 0.005, *** *p* < 0.0005. Significance was determined using the Mann–Whitney U-test, an agitator-horizontal platelet shaker LPS-A20 (LABTRON, Comberlay, UK), and a Centrifugation–Megafuge 16R (Thermo Fisher Scientific, Waltham, MA, USA).

The physiological concentrations of the following salts in plasma are NaCl 136–145 mM, KCL 3–5 mM, and CaCl_2_ 2–2.5 mM. Factor VIII with thrombin, dissolved in the indicated salt solution (15 mM), was set in motion via 10 min centrifugation (500 g) at room temperature, and then the level of factor VIII was measured via Western blot. Comparison of the effect elicited by each of the above salts, dissolved at the same concentration in double-distilled water (DDW), demonstrated that only calcium prevented the degradation of factor VIII. The protective effect persisted even when the solutions were centrifuged (Figure 1A) followed by 10 min agitation at 30 RPM (Figure 1B). As all the aforementioned salts have Cl^−^ ions, the comparison indicates that Ca^+2^ ions and not Cl^−^ ions are responsible for the stabilization effect. Calcium dissolved in DDW was found to prevent factor VIII degradation in physiologic plasma concentrations (1.5 mM, 3 mM), in a dose-dependent manner. Relative protein levels were quantified via densitometry and expressed as optical density.

### 3.2. Calcium Levels in the Microcirculation of Skeletal Striated Muscles Are Elevated following Movement

**Figure 2:**
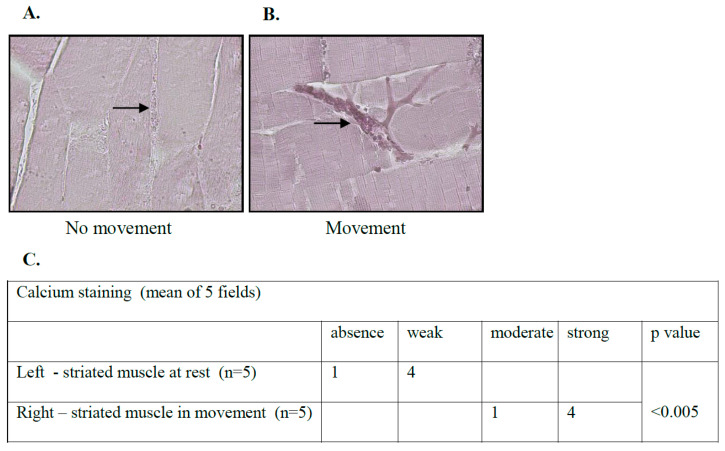
Calcium levels in the microcirculation of skeletal striated muscles are elevated following movement. ICR mice (no specific genetic background) were put to sleep following isoflurane anesthesia for 15 min. The study group (n = 5) continued regular activity in the cage. Both groups were sacrificed, and the hind legs’ thigh striated muscles were studied using Alizarin Red staining specific for calcium ions. A significant increase in the calcium level in the microcirculation was observed in mice in the condition of movement relative to resting mice ((**A**,**B**) black arrows, (**C**)-statistical evaluation), enabling the prevention of factor VIII degradation in normal physiology. Representative images were captured with a Nikon E995 digital camera (Nikon, Tokyo, Japan), original magnification, ×10. Contiguous table shows calcium staining intensity in striated muscles. Significance was determined using the Mann–Whitney U-test (absence + week groups were compared to moderate + strong groups).

ICR mice (with no specific genetic background) were put to sleep following isoflurane anesthesia for 15 min. The study group (n = 5) continued regular activity in the cage. Both groups were sacrificed, and the striated muscles of the hind legs’ thighs were investigated using Alizarin Red staining specific for calcium ions. A significant increase in calcium levels in the microcirculation was observed in mice during movement compared to resting animals (Figure 2), enabling the prevention of factor VIII degradation in normal physiology. We found no difference in the staining level in other organs that were analyzed, including the heart muscle, small intestine, brain, kidney, liver, and lungs implying that physiologically higher calcium levels in the microcirculation occur specifically in contracting skeletal striated muscles to prevent bleeding. 

### 3.3. Calcium Supplementation in Drinking Water Raises the Level of Factor VIII in the Blood and Skeletal Striated Muscles

**Figure 3:**
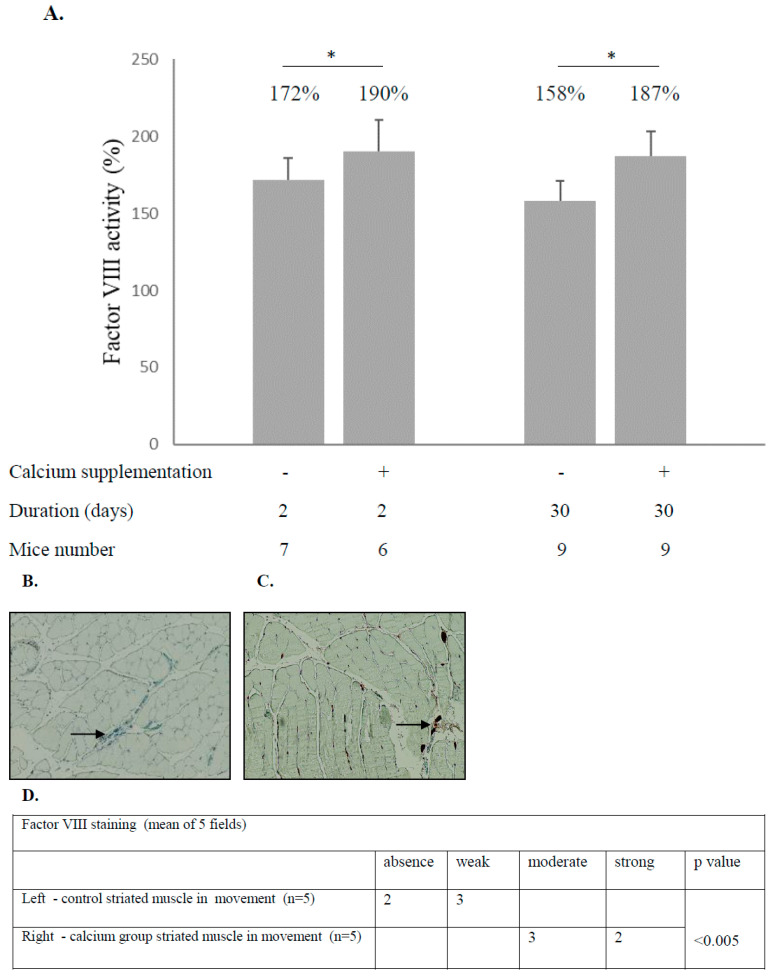
Calcium supplementation in drinking water raises the level of Factor VIII in the blood and skeletal striated muscles of mice. Seven- to eight-week-old male ICR mice were divided into two groups. One group received the recommended daily dose of calcium gluconate (Solgar, water-soluble) supplementation in drinking water (7.5 mM), and the control group received drinking water without additives. The experiment was conducted twice with two different mouse groups. The first experiment lasted for two days and the second for one month. At the end of the experiments, blood was drawn, and the mice were sacrificed. In mice that drank water with calcium, factor VIII levels in plasma were higher than in mice that drank water without calcium supplementation (**A**). In addition, in mice that drank water with calcium, factor VIII levels in the hind legs’ thigh striated muscle microcirculation were also higher compared to levels in the control group ((**B**,**C**) black arrows, (**D**)-statistical evaluation). The result of (**A**). represents mean ± SEM. Significance was determined using the Mann–Whitney U-test (* *p* < 0.05). The significance of (**B**,**C**) was determined using the U-test (absence + week groups were compared to moderate + strong groups). Results were similar after two days and after 1 month of experiments (**A**–**C**).

Seven- to eight-week-old male ICR mice were divided into two groups. One group received the recommended daily dose of calcium gluconate (Solgar, water-soluble, Leonia, NJ, USA) supplementation in drinking water (7.5 nM), and the control group received drinking water without additives. The experiment was conducted twice with two different mice groups. The first experiment lasted for two days, while the second lasted for one month. At the end of the experiments, blood was drawn and the mice were sacrificed. In the animals that drank water with calcium, factor VIII levels in plasma were higher than mice that drank water without calcium supplementation (190% ± 21 vs. 172% ± 14, 187% ± 16 vs. 158 ± 13, *p* < 0.05 and *p* < 0.001, respectively). In addition, in mice that drank water with calcium, factor VIII levels in the hind legs’ thigh striated muscle microcirculation were also higher compared to levels in the control group. Similar results were obtained after two days and 1 month of experiments (Figure 3A–C).

### 3.4. Calcium Solution Does Not Prevent Factor IX Degradation during Movement

**Figure 4:**
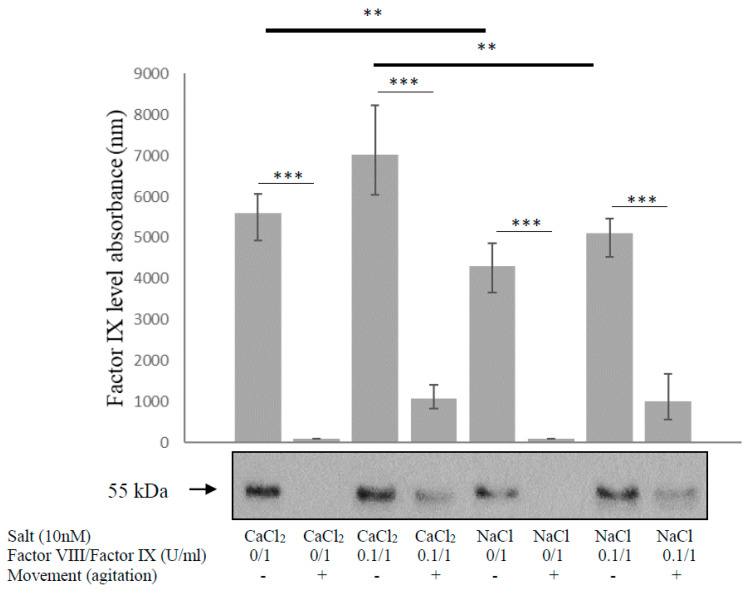
Calcium solution does not prevent factor IX degradation in movement. Factor IX in the indicated solution was placed in the agitator for 10 min (30 RPM) at room temperature, and then the level of factor IX was measured using Western blot. Addition of factor VIII prevented factor IX degradation, while movement enhanced its degradation. Although calcium solution in comparison to NaCl solution prevented degradation of factor IX in the resting condition (thick horizontal lines), the effect was similar in movement and did not prevent factor IX degradation (thin horizontal lines). Relative protein levels in Western blots were quantified via densitometry analysis (upper panel). Assays were conducted in triplicate. The results represent mean and range. ** *p* < 0.005, *** *p* < 0.0005. Significance was determined by the Mann–Whitney U-test and Agitator-horizontal platelet shaker LPS-A20 (LABTRON, Comberlay, UK).

We previously demonstrated that movement enhanced the degradation of factor IX, but in contrast to factor VIII, the effect was independent of thrombin. Factor VIII was found to protect factor IX from degradation. The current study was designed to explore whether calcium had a protective effect on factor IX. Factor IX in the indicated solution was placed in the agitator for 10 min at room temperature (30 RPM), and then the level of factor IX was evaluated via Western blot. Addition of factor VIII prevented factor IX degradation, while movement resulted in its enhancement. Although calcium solution in comparison to NaCl solution prevented degradation of factor IX in the resting condition, the effect was similar in movement, and did not prevent factor IX degradation (Figure 4). Relative protein levels were quantified using densitometry and expressed as optical density. 

## 4. Discussion

In the current study, the calcium salt solution at physiological concentrations was found to preserve factor VIII from degradation, unlike Na^+^ and K^+^ ions at similar concentrations. Interestingly, movement appeared to induce the release of calcium into the microcirculation (as demonstrated by specific staining), which could contribute to prevention of factor VIII degradation. Based on this finding, we hypothesized that calcium supplementation in mouse intake would increase blood calcium levels and reduce factor VIII degradation in striated muscles during movement. Indeed, calcium supplementation in drinking water, at a recommended daily dose, led to an elevation of factor VIII levels in the plasma and striated muscles of the mice. Given such promising results, this approach needs to be investigated as a potential therapeutic option for patients with hemophilia A.

Furthermore, as we previously reported [8], movement was found to be associated with enhanced degradation of factor IX. The effect was observed without addition of thrombin, although traces of thrombin are present in the factor IX preparation. In addition, factor VIII appeared to protect factor IX against degradation and the effect persisted during movement [8]. As demonstrated in our present study, unlike factor VIII, calcium ions had no significant protective effect on factor IX during motion.

Calcium had been previously shown to stabilize the factor VIII–vWF complex and prevent factor VIII degradation by thrombin [9,10]. Our current data demonstrate that the protective effect of calcium is preserved following movement. Thus, for patients with hemophilia, calcium supplementation might be beneficial owing to attenuation of factor VIII degradation. However, an increase in factor VIII levels in the circulation, associated with calcium supplementation, may pose a hazard and predispose to thrombosis. In the study by Bristow et al., 100 postmenopausal women were randomized to 1 g/d of calcium (recommended daily supplementation dose) or a placebo containing no calcium. Four hours following supplement intake, an increase in blood coagulation was observed, as indicated by a significantly shorter time to clot formation evaluated with a thromboelastographic (TEG) assay [14]. In another randomized placebo-controlled trial, including 36,282 community-dwelling postmenopausal women with 7 years of follow-up, calcium and vitamin D (1 g calcium and 400 IU vitamin D daily) supplementation appeared to elevate the risk of myocardial infarction [relative risk 1.21 (95% confidence interval 1.01 to 1.44), *p* = 0.04], stroke [1.20 (1.00 to 1.43), *p* = 0.05], and the composite of myocardial infarction or stroke [1.16 (1.02 to 1.32), *p* = 0.02] [15]. Furthermore, among 27,685 subjects (25–87 years) participating in Tromsø 4 (1994–1995) and 8547 individuals participating in Tromsø 5 (2001–2002) surveys, where total calcium and PTH were measured, 712 cases of venous thromboembolism (VTE) were documented during a median follow-up of 15 years. Subjects with high serum levels of both calcium and PTH (calcium ≥2.45 mmol/L and PTH ≥4.0 pmol/L) had an increased risk of VTE compared to that in subjects with normal calcium and PTH values (multivariable HR: 1.78, 95% CI: 1.12–2.84) [16]. These studies support a correlation between calcium levels and enhanced activation of the coagulation system. Our study suggests that calcium intake leads to an increased factor VIII level, which is known to contribute to both arterial and venous thrombosis [17,18,19,20].

## 5. Conclusions

In conclusion, the present study has demonstrated that the protective effect of calcium on factor VIII degradation persists during movement. Supplementation of oral calcium increases factor VIII levels and may be evaluated as a potential supportive therapy in hemophilia patients.

## Data Availability

The current study’s data are available from the corresponding author on reasonable request.
